# A randomized controlled phase Ia clinical trial to evaluate the safety, tolerability and pharmacokinetic characteristics of tofacitinib tartrate cream (HZ-J001) in healthy Chinese subjects

**DOI:** 10.3389/fphar.2026.1821901

**Published:** 2026-05-07

**Authors:** Kunhong Deng, Jie Huang, Qian Wu, Jinlian Xie, Xiaoyan Yang, Shuang Yang, Li Liu, Yafang Gong, Wanyao Shen, Ling Cai, Guoping Yang, Chengxian Guo

**Affiliations:** 1 Center of Clinical Pharmacology, The Third Xiangya Hospital, Central South University, Changsha, China; 2 Xiangya School of Pharmaceutical Sciences, Central South University, Changsha, China; 3 Gynecology Department, The Third Xiangya Hospital, Central South University, Changsha, China; 4 Hubei Chengtian Pharmaceutical Co., Ltd., Tianmen, China

**Keywords:** JAK inhibitor, pharmacokinetics, phase I trial, safety, tofacitinib

## Abstract

**Introduction:**

HZ-J001 is a novel topical tofacitinib tartrate cream designed to optimize local pharmacokinetics (PKs) and minimize systemic adverse events (AEs). This study aimed to evaluate the safety, tolerability, and PK characteristics of HZ-J001 cream in healthy Chinese subjects.

**Methods:**

This randomized, double-blind, placebo-controlled Phase Ia trial included both single-ascending-dose (SAD) and multiple-dose (MD) phases. Healthy subjects were enrolled into 5 sequential cohorts (10 HZ-J001 and 2 placebo per cohort). SAD Cohorts 1-3 received single doses of HZ-J001 cream at strengths of 1.0%, 1.5%, and 2.0%, respectively, each applied to a fixed area of 20% body surface area (BSA) (3,200 cm^2^). SAD Cohort 4 received the 2.0% strength applied to a larger area of 30% BSA (4,800 cm^2^). MD Cohort 5 received the 2.0% strength to 30% BSA (4,800 cm^2^) twice daily for 8 days and once on Day 9 (17 doses in total). A fixed amount of 3 mg cream/cm^2^ was applied for all subjects. The PK parameters and treatment-emergent adverse events (TEAEs) were evaluated.

**Results:**

A total of 61 subjects were enrolled and 60 received study drug. HZ-J001 cream was well-tolerated and no serious TEAEs or discontinuations occurred. The most common TEAEs by system organ class were investigations. Local tolerability was acceptable, with only mild application-site reactions reported. Systemic exposure following single-dose administration was low (C_max_ and AUC_0-t_ were 0.09–0.16 ng/mL and 7.24–12.18 h·ng/mL, respectively). Although drug accumulation occurred after repeated dosing (accumulation ratios: 15.71 for C_max_ and 28.80 for AUC_0-τ_), overall systemic exposure remained low.

**Conclusion:**

HZ-J001 cream demonstrated a favorable safety profile and a PK characteristic of minimal systemic exposure in healthy subjects, supporting its further development as a topical therapy for atopic dermatitis.

## 1 Introduction

Atopic dermatitis (AD) is a chronic, relapsing inflammatory skin disease characterized predominantly by intense pruritus that substantially impairs patients’ quality of life (Langan et al., 2020). It affects approximately 20% of children and 10% of adults globally, representing a major public health burden (Xue and Yao, 2025). The pathogenesis of AD reflects a complex interplay among genetic predisposition, epidermal barrier dysfunction, immune dysregulation, and epigenetic modifications (Langan et al., 2020). Therapeutic strategies focus on restoring barrier function, emollient therapy, and controlling inflammation (Wollenberg et al., 2018). The choice of anti-inflammatory treatment depends on disease severity, with systemic immunomodulatory therapies reserved for severe cases and topical treatments used for milder disease (Wollenberg et al., 2018; Mazgaj et al., 2025). Current topical options include emollients, topical corticosteroids, and calcineurin inhibitors (CNIs) (Eichenfield et al., 2014), as well as more recently approved targeted therapies. These encompass topical phosphodiesterase 4 (PDE4) inhibitors (e.g., crisaborole and roflumilast), nonsteroidal topical aryl hydrocarbon receptor (AhR) agonists (e.g., tapinarof), and topical Janus kinase (JAK) inhibitors (e.g., ruxolitinib and delgocitinib) (Gallagher et al., 2025; Meledathu et al., 2025). Despite these significant pharmacological advancements, the clinical management of AD remains challenging. Many current topical formulations are still constrained by suboptimal local tolerability, insufficient long-term efficacy, or restricted application scenarios (Li et al., 2021; Nakashima et al., 2022; Mazgaj et al., 2025). Consequently, there remains a compelling need to continuously develop and optimize novel topical treatments with improved efficacy and favorable safety profiles.

JAK inhibitors are a novel class of immunomodulatory agents that block JAK enzymatic activity and thus attenuate multiple cytokine-driven pathways (Xue et al., 2023). They have emerged as therapeutic targets for immune-mediated dermatologic diseases (Mazgaj et al., 2025). Tofacitinib is a JAK inhibitor that predominantly targets JAK1 and JAK3 (Bissonnette et al., 2016). Its efficacy has been demonstrated in multiple immune-mediated diseases, including rheumatoid arthritis (Zhu et al., 2025), ulcerative colitis (Lin et al., 2024), psoriatic arthritis (Ighani et al., 2020), and ankylosing spondylitis (Burhanuddin et al., 2025). Additionally, accumulating evidence supports its therapeutic potential in AD (Zhang et al., 2021). The mechanism by which tofacitinib improves AD likely involves inhibition of Janus kinase-signal transducer and activator of transcription (JAK-STAT) signaling, thereby blocking the phosphorylation and activation of STATs and subsequent inflammatory cytokine signaling (e.g., IL-4, IL-13, and IL-31) essential for AD pathogenesis (Miot et al., 2023; Mazgaj et al., 2025).

Currently, oral formulations of tofacitinib, including tablets, extended-release tablets, and oral solution, have been approved for the treatment of rheumatoid arthritis, psoriatic arthritis, ulcerative colitis, and polyarticular juvenile idiopathic arthritis (Nakashima et al., 2022). However, systemic administration is associated with increased risks of serious infections, malignancy, cardiovascular events, and thrombosis (Mazgaj et al., 2025), which limits its long-term use. These concerns have prompted interest in the development of topical formulations, which may provide effective local treatment while minimizing systemic exposure. Although other topical JAK inhibitors (such as ruxolitinib and delgocitinib) have been recently approved for AD (Dhillon, 2020; Gallagher et al., 2025), there remains a compelling clinical need for more diverse, highly effective, and well-tolerated options, particularly for patients who exhibit suboptimal responses or intolerance to current therapies. In this context, the continued development of topical tofacitinib is strongly justified by its distinct pharmacological profile. Tofacitinib is a preferential JAK1 and JAK3 inhibitor, which differs fundamentally from the target spectrum of ruxolitinib (a JAK1/JAK2 inhibitor) or delgocitinib (a pan-JAK inhibitor) (Nakashima et al., 2022). This distinct pathway blockade may translate into a differentiated efficacy and safety profile (Huang et al., 2022). In addition, the extensive global clinical experience with oral tofacitinib provides a remarkably well-characterized foundation of efficacy and safety (Wollenhaupt et al., 2019).

Several early-phase clinical studies have evaluated topical tofacitinib formulations, including ointments (Ports et al., 2013; Bissonnette et al., 2016; Bagherani and Smoller, 2017; Putterman and Castelo-Soccio, 2018; Purohit et al., 2019; Berbert Ferreira et al., 2023; Chikhalkar et al., 2024), creams (Cheng et al., 2018), gels (Kerkemeyer et al., 2021),and solutions (Ports et al., 2015; Kerkemeyer et al., 2021), mainly for the treatment of AD (Bissonnette et al., 2016; Purohit et al., 2019), psoriasis (Ports et al., 2013; Ports et al., 2015; Bagherani and Smoller, 2017; Berbert Ferreira et al., 2023), and alopecia areata (Cheng et al., 2018; Putterman and Castelo-Soccio, 2018; Kerkemeyer et al., 2021; Chikhalkar et al., 2024). In early-phase studies, topical tofacitinib has shown encouraging results, particularly in terms of local therapeutic efficacy and reduced systemic exposure. Nevertheless, no topical formulation of tofacitinib has yet received regulatory approval, and its clinical availability remains limited, underscoring the need for further optimization to enhance skin penetration, achieve adequate local drug concentrations, and ensure long-term safety.

HZ-J001 cream containing tofacitinib tartrate was developed by Hubei Humanwell Chengtian Pharmaceutical Co., Ltd. as a novel topical cream formulation. This drug was designed by modifying the indication, formulation, route of administration, salt form, and manufacturing process of oral tofacitinib citrate. Comparative dermal permeation studies of different tofacitinib salt forms demonstrated that HZ-J001 exhibits favorable local pharmacokinetic (PK) properties. It achieves higher drug concentrations at lesional sites to ensure efficacy while limiting percutaneous absorption into the systemic circulation, thereby reducing the risk of systemic adverse event (AEs) associated with systemic exposure.

Currently, the therapeutic potential of topical JAK inhibitors is broadly recognized across various inflammatory dermatoses (Miot et al., 2023). Accordingly, the intended clinical applications of HZ-J001 cream encompass a broad spectrum of immune-mediated skin conditions, including AD, vitiligo, and alopecia areata. Among these, AD represents a key target indication of HZ-J001 cream for future patient explorations. This is primarily driven by the central role of the JAK-STAT signaling pathway (particularly via IL-4, IL-13, and IL-31) in AD pathogenesis, the immense global disease burden affecting both pediatric and adult populations, and the urgent clinical demand for safer, long-term topical anti-inflammatory agents (Nakashima et al., 2022). Furthermore, the clinical efficacy and safety of topical JAK inhibitors have been clearly demonstrated by the successful regulatory approvals of other pioneering topical JAK inhibitors for AD, such as delgocitinib in Japan (Dhillon, 2020) and ruxolitinib in the United States (Gallagher et al., 2025). This established post-marketing experience firmly positions JAK inhibition as a proven therapeutic strategy for AD.

This study is a randomized, double-blind, placebo-controlled phase Ia clinical trial designed to evaluate the safety, tolerability, and PK characteristics of single and multiple topical applications of HZ-J001 cream in healthy adult subjects. Building upon the foundational safety and PK profiles established in this trial in healthy subjects, the therapeutic potential of HZ-J001 in AD and other intended dermatologic indications will be extensively explored in subsequent clinical trials.

## 2 Methods

### 2.1 Ethics statement

This Phase Ia study was conducted at the Third Xiangya Hospital of Central South University in accordance with the Declaration of Helsinki and Good Clinical Practice (GCP). The study protocol was reviewed and approved by the Ethics Committee of the Third Xiangya Hospital of Central South University (Approval No. 24012) prior to study initiation and was registered with the Center for Drug Evaluation (CDE) of the National Medical Products Administration (NMPA) in March 2024 (Registration No. CTR20240757; http://www.chinadrugtrials.org.cn). Written informed consent was obtained from all participants prior to their involvement in the study.

### 2.2 Study design

This study employed a randomized, double-blind, placebo-controlled design, incorporating both single-ascending-dose (SAD) and multiple-dose (MD) phases. A total of 60 healthy subjects were planned to be enrolled and randomized into 5 cohorts (n = 12 per cohort), each comprising 10 subjects receiving HZ-J001 cream and 2 receiving placebo. Randomization sequences were generated by an independent statistician, who was not otherwise involved in the study, using a block randomization method in SAS software (version 9.4). Allocation concealment was ensured through the use of sequentially numbered, sealed, opaque envelopes maintained by a designated staff member. The investigators, subjects, and data analysts remained blinded to the treatment allocation until the official unblinding of the trial. The sample size for this study was not determined based on formal statistical power calculations. Instead, it was established based on industry conventions and empirical rules of thumb for early-phase safety and PK evaluations (e.g., 10 to 12 subjects per dose cohort). Patients or the public were not involved in the design, conduct, or reporting of this trial.

Detailed study cohorts and dosing schedules for HZ-J001 cream administration are summarized in Table 1. All administrations used a fixed amount (formulation weight) of 3 mg cream/cm^2^. The preferred application sites, in order of priority, included the back, abdomen, thighs, and upper arms, and the selected site remained consistent for each subject throughout the study. The applied body surface area (BSA) was calculated based on a reference body weight of 60 kg using the formula:

$${\mathrm{Log}}_{10}S=0.698\times {\mathrm{Log}}_{10}W+0.8762$$

where S is BSA (cm^2^) and W is body weight (g). The calculated total BSA was approximately 1.6 m^2^, corresponding to 20% BSA of 3,200 cm^2^ and 30% BSA of 4,800 cm^2^.

**TABLE 1:** Study cohorts and dosing schedules for HZ-J001 cream administration.

Cohort	Strengths	Number of healthy subjects	Dosing day(s)	Application area and amount
SAD cohort 1	1.0%	12(HZ-J001: 10, placebo: 2)	D1	3,200 cm^2^, 3 mg/cm^2^
SAD cohort 2	1.5%	12(HZ-J001: 10, placebo: 2)	D1	3,200 cm^2^, 3 mg/cm^2^
SAD cohort 3	2.0%	12(HZ-J001: 10, placebo: 2)	D1	3,200 cm^2^, 3 mg/cm^2^
SAD cohort 4	2.0%	12(HZ-J001: 10, placebo: 2)	D1	4,800 cm^2^, 3 mg/cm^2^
MD cohort 5	2.0%	12(HZ-J001: 10, placebo: 2)	D1-D8 (twice daily), D9 (once daily)	4,800 cm^2^, 3 mg/cm^2^

SAD, single-ascending-dose; MD, multiple-dose.

The SAD phase consisted of 4 cohorts to evaluate the impact of different drug strengths and application BSA. SAD Cohorts 1-3 received single doses of HZ-J001 cream at strengths of 1.0%, 1.5%, and 2.0%, respectively, each applied to a fixed area of 20% BSA (3,200 cm^2^). SAD Cohort 4 received the 2.0% strength applied to a larger area of 30% BSA (4,800 cm^2^). Subjects in the SAD phase received a single dose under fasting conditions, and the applied cream was removed 24 h (±30 min) after application.

The MD phase included one cohort (Cohort 5), which received the 2.0% strength applied to 30% BSA (4,800 cm^2^) for a total of 17 doses. From Day 1 to Day 8, the cream was applied twice daily at 12-h intervals (±30 min), and each application was removed 12 h (−30 min) later. On the morning of Day 9, a final dose was applied and wiped off 24 h (±30 min) later. The initial dose on Day 1 and the final dose on the morning of Day 9 were administered under fasting conditions, whereas all other doses followed standard meal schedules. During the drug application period (SAD: 24 h; MD: Days 1–9), bathing or showering was not permitted. Subjects were discharged on Day 8 for the SAD cohorts and on Day 16 for the MD cohort.

### 2.3 Study participants

Healthy Chinese adults aged 18–45 years with a body mass index (BMI) of 19.0–26.0 kg/m^2^ (with body weight requirements of ≥50.0 kg for males and ≥45.0 kg for females) were enrolled in this study. Key exclusion criteria included a history of clinically significant organ dysfunction or dermatologic diseases; known hypersensitivity to any drug component or documented allergic predisposition; a positive skin scratch test or compromised skin integrity at the application sites; and any clinically significant abnormalities identified by vital signs assessment, physical examination, laboratory tests, 12-lead electrocardiography, or imaging examinations. Additional exclusion criteria included the use of any medication within 14 days prior to dosing, recent blood donation, a history of substance misuse, alcohol abuse, significant tobacco use, and pregnancy or lactation.

### 2.4 Study drugs

The investigational product, HZ-J001 cream, was supplied by Hubei Humanwell Chengtian Pharmaceutical Co., Ltd. Three formulation strengths were evaluated: 1.0% (10 g containing 0.1 g tofacitinib tartrate; batch No. C8023001), 1.5% (10 g containing 0.15 g tofacitinib tartrate; batch No. C8123001), and 2.0% (10 g containing 0.2 g tofacitinib tartrate; batch No. C8223001). To maintain the double-blind design, the placebo comparator was formulated to be identical to the active HZ-J001 cream in appearance, color, odor, and texture, lacking only the active pharmaceutical ingredient (batch No. 20230401). The vehicle formulation of the placebo cream consisted of polyethylene glycol-7 stearate, cetyl alcohol, dimethicone, light liquid paraffin, propylparaben, methylparaben, and purified water. The excipient composition of the HZ-J001 cream was completely identical to that of the placebo. To guarantee dosing accuracy and treatment fidelity, all applications and removals of the study drug were performed exclusively by trained clinical study staff. The exact amount of cream administered was strictly controlled by weighing the containers before and after each application.

### 2.5 Safety and tolerability

Safety and tolerability were assessed through continuous monitoring of treatment-emergent adverse events (TEAEs) and systematic evaluations of vital signs (blood pressure, pulse rate, respiratory rate, and body temperature), physical examinations, clinical laboratory tests (hematology, urinalysis, serum biochemistry, and coagulation profiles), infectious disease screening, serum pregnancy testing for women of childbearing potential, 12-lead electrocardiography, and local skin tolerability. Local skin tolerability was assessed by examination of the application sites, including evaluation of predefined subjective symptoms and objective cutaneous findings. Detailed grading criteria for local skin tolerability are provided in Supplementary Table S1.

All TEAEs were coded by the investigators using the Medical Dictionary for Regulatory Activities (MedDRA, version 27.1). The severity of TEAEs was graded according to the National Cancer Institute Common Terminology Criteria for Adverse Events (NCI CTCAE, version 5.0). For continuous variables, fluctuations within the normal reference range were not considered TEAEs. An abnormality was recorded as a TEAE only if the value fell outside the normal reference laboratory range and was determined to be clinically significant by the investigator. All TEAEs were actively followed until resolution, return to baseline, or stabilization.

### 2.6 PK evaluations and bioanalysis

Blood samples for PK evaluation were collected according to predefined sampling schedules. In SAD Cohort 1, blood samples were obtained at pre-dose, and at 0.5, 1, 1.5, 2, 2.5, 3, 5, 7, 7.5, 8, 8.5, 9, 12, 24 (Day 2), 48 (Day 3), 72 (Day 4), 96 (Day 5), 120 (Day 6), 144 (Day 7), and 168 (Day 8) hours post-dose. Given the consistently low plasma concentrations observed in SAD Cohort 1, the sampling schedule for subsequent SAD cohorts was optimized to the following time points: pre-dose and at 4, 8, 12, 16 (Day 2), 20 (Day 2), 24 (Day 2), 30 (Day 2), 36 (Day 2), 42 (Day 3), 48 (Day 3), 72 (Day 4), 96 (Day 5), and 168 (Day 8) hours post-dose. For the MD cohort (Cohort 5), blood samples was collected at pre-dose and at 4, 8, and 12 h post-dose on Day 1 (morning doses); pre-dose on Days 7, 8, and 9 (morning doses); pre-dose on Day 8 (evening dose); and at 4, 8, 12, 16 (Day 10), 20 (Day 10), 24 (Day 10), 30 (Day 10), 36 (Day 10), 42 (Day 11), 48 (Day 11), 72 (Day 12), 96 (Day 13), and 168 (Day 16) hours after the final dose on Day 9.

Approximately 4 mL of blood per sample was collected into EDTA-K_2_ anticoagulant tubes and centrifuged at 2000 × g for 10 min at 4 °C. The resulting plasma was stored at ≤−60 °C until analysis. Plasma concentrations of tofacitinib were quantified using a validated liquid chromatography-tandem mass spectrometry (LC-MS/MS) method. A stable isotope-labeled compound, tofacitinib-d3, was utilized as the internal standard (IS). Sample preparation was performed via liquid-liquid extraction. An aliquot of 100 μL of plasma sample was extracted with 600 μL of ethyl acetate, followed by centrifugation, nitrogen drying of the supernatant, and reconstitution in a 20% acetonitrile solution containing 1% formic acid. The assay demonstrated a linear calibration range of 0.01–10.0 ng/mL, with a lower limit of quantification (LLOQ) of 0.01 ng/mL. Comprehensive analytical validation was conducted in accordance with current regulatory guidelines. The intra- and inter-batch precision values (coefficient of variation, CV%) were 4.3% and 3.9%, respectively. The overall assay accuracy (expressed as relative error), both intra-batch and inter-batch, consistently remained below 5.3%. The mean extraction recoveries for low-, medium-, and high-concentration quality control samples were 83.3%, 81.5%, and 84.6%, respectively. Evaluation of matrix effects yielded an IS-normalized matrix factor ranging from 0.97 to 0.99 (CV%≤3.1%). Furthermore, tofacitinib in human plasma was confirmed to be stable under all relevant conditions, including stability at room temperature for 26 h, four freeze-thaw cycles, post-preparative autosampler stability at 4 °C for 121 h, and long-term storage at ≤ −60 °C for at least 108 days.

### 2.7 Statistical analyses

The statistical analyses were conducted based on four predefined analysis sets. The full analysis set (FAS) used for demographic characteristics encompassed all randomized subjects who received at least one dose of the study drug. The safety set (SS) included all subjects who received at least one dose of the study drug and had at least one post-dose safety assessment. The pharmacokinetic concentration set (PKCS) comprised all subjects who received at least one dose of the study drug and provided at least one valid post-dose measurable drug concentration. Finally, the pharmacokinetic parameter set (PKPS) included all subjects who received at least one dose of the study drug and had sufficient concentration data to calculate at least one primary PK parameter, which was used for the descriptive summary of PK parameters. Subjects were excluded from the PKPS if they had major protocol deviations that could significantly affect the PK evaluation or render PK parameters incalculable, or if they used concomitant medications known to interfere with the PK profile of the study drug. Regarding the handling of missing data, no imputation was performed, and all statistical summaries were based solely on observed data.

PK parameters were estimated using a noncompartmental analysis (NCA) in WinNonlin version 8.3 (Pharsight Corporation, Mountain View, CA, USA). Plasma concentrations below the lower limit of quantification (BLQ) occurring prior to the time of maximum concentration (T_max_), including pre-dose samples, were set to zero. Any BLQ values occurring after T_max_ in the elimination phase were treated as not detectable (missing data) and were excluded from the NCA parameters calculations. Key parameters analyzed after the first dose included the area under the concentration versus time curve from zero to the last quantifiable concentration time (AUC_0-t_), the area under the concentration versus time curve from zero to infinity (AUC_0-∞_), maximum concentration (C_max_), T_max_, and terminal elimination half-life (t_1/2_).

For the MD cohort, the dosing interval (τ) was 12 h. The area under the concentration versus time curve during the dosing interval on Day 1 was strictly defined as AUC_0−τ_. Following MD administration, parameters included the area under the concentration versus time curve during the dosing interval at steady state (AUC_ss,0-τ_), minimum (C_ss,min_) and maximum (C_ss,max_) steady-state concentrations, average steady-state concentration (C_ss,av_), time to C_ss,max_ (T_ss,max_), and accumulation ratios (R_ac_). The R_ac_ for C_max_ was calculated as C_ss,max_/C_max_, and R_ac_ for AUC_0-τ_ was calculated as AUC_ss,0-τ_/AUC_0-τ_, where C_max_ and AUC_0-τ_ represent the values observed within 12 h after the first dose on Day 1, whereas C_ss,max_ and AUC_ss,0-τ_ represent the values observed within 12 h after the last dose on Day 9.

Descriptive statistics were applied to all PK parameters, and mean plasma concentration-time profiles were generated to characterize the PK characteristics. The effects of age, sex, and body weight on PK parameters in each cohort were also explored.

All statistical analyses were performed using SAS version 9.4 (SAS Institute Inc., Cary, NC, USA). Two-sided tests were employed for statistical comparisons. Parameter estimates are presented with two-sided 95% confidence intervals (CIs) and descriptive results are presented as counts, percentages, means, and standard deviations (SD). Dose proportionality (SAD Cohort 1–4) for C_max_, AUC_0-t_ and AUC_0-∞_ was evaluated using a power model. Separate proportionality analyses were conducted for strength escalation (SAD Cohort 1–3) and application area escalation (SAD Cohort 3–4) to assess the respective proportionality relationships. These escalation scenarios ultimately translated to an increase in the total administered dose. The proportionality relationship was analyzed using a log-transformed power model, expressed as the following linear regression equation:

$$\ln\left(\text{PK}\right)=\beta_{0}+\beta \times \ln\left(\text{Dose}\right)+\varepsilon$$

where PK is the PK parameter, Dose is the administered dose, β_0_ is the intercept, *β* is the estimated slope, and *ε* is the random error term. Proportionality was concluded if the 90% CI for β fell entirely within the pre-defined decision interval (β_L_-β_U_). The standard interval was constructed as β_L_ = 1 + lnθ_L_/ln_γ_ and β_U_ = 1 + lnθ_U_/ln_γ_, where γ represents the ratio of the highest to the lowest total dose within the specific evaluation scenario. The equivalence limits were set as θ_L_ = 0.8 and θ_U_ = 1.25 for AUC_0-t_ and AUC_0-∞_, whereas wider limits of θ_L_ = 0.7 and θ_U_ = 1.43 were prespecified for C_max_.

Steady-state attainment in the MD cohort was assessed by regression analysis of trough concentrations collected prior to the morning doses on Days 7, 8, and 9. Safety analyses were performed descriptively in all subjects who received at least one dose of the study drug.

## 3 Results

### 3.1 Demographic characteristics

**FIGURE 1:**
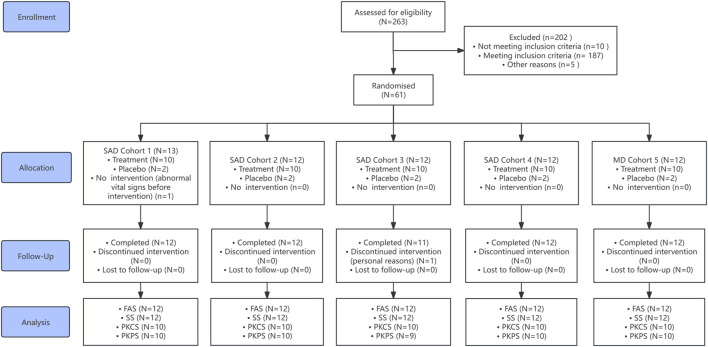
Disposition process of subjects SAD, single-ascending-dose; MD, multiple-dose; FAS, full analysis set; SS, safety set; PKCS, pharmacokinetic concentration set; PKPS, pharmacokinetic parameter set.

This study was conducted between March 2024 and 1 August 2024. A total of 263 subjects were screened, and 61 subjects (51 assigned to HZ-J001 and 10 to placebo) were enrolled in the study. The disposition process of subjects is shown in Figure 1. In the SAD Cohort 1, one subject (assigned to the HZ-J001 group) was withdrawn prior to drug administration due to abnormal pre-dose vital signs and did not receive the study drug. This subject was excluded from all analysis sets and was replaced by a substitute subject. In the SAD Cohort 3, one subject (HZ-J001 group) withdrew prematurely due to personal reasons, resulting in early discontinuation of study drug administration. Only four planned PK samples were collected from this subject, and due to the lack of any calculable PK parameters, the subject was excluded from the PKPS. The remaining 59 subjects completed the study. Baseline demographic characteristics are summarized in Table 2. The demographic and baseline characteristics were balanced and comparable across all cohorts. The majority of subjects were male (male: female = 52:8). The overall population had a mean age of 27.80 ± 6.87 years and a mean body weight of 62.88 ± 7.32 kg.

**TABLE 2:** Demographic characteristics of subjects.

Characteristics	SAD Cohort1 (N = 10)	SAD cohort 2 (N = 10)	SAD cohort 3 (N = 10)	SAD cohort 4 (N = 10)	MD cohort 5 (N = 10)	Placebo (N = 10)	Total (N = 60)
Male/Female	5/5	8/2	10/0	10/0	10/0	9/1	52/8
Age (year)	29.40 ± 7.86 (20.00,42.00)	23.90 ± 5.20 (18.00,35.00)	30.80 ± 7.42 (18.00,38.00)	27.30 ± 6.65 (18.00,36.00)	26.70 ± 6.29 (19.00,41.00)	28.70 ± 7.02 (20.00,38.00)	27.80 ± 6.87 (18.00,42.00)
Body weight (kg)	62.15 ± 8.17 (49.30,75.80)	59.76 ± 6.23 (51.60,70.50)	61.85 ± 8.97 (53.20,82.10)	63.78 ± 6.28 (53.20,74.70)	66.87 ± 7.53 (60.70,84.20)	62.87 ± 6.13 (56.90,74.80)	62.88 ± 7.32 (49.30,84.20)
Height (cm)	165.60 ± 7.06 (154.00,174.50)	164.80 ± 3.54 (157.50,170.50)	168.65 ± 7.09 (160.50,181.50)	168.45 ± 4.60 (161.50,175.50)	171.55 ± 5.98 (160.00,180.00)	165.95 ± 6.80 (155.50,179.00)	167.50 ± 6.19 (154.00,181.50)
BMI (kg/m2)	22.58 ± 1.73 (20.30,25.30)	21.98 ± 1.92 (19.40,25.30)	21.63 ± 1.46 (20.00,24.90)	22.50 ± 2.15 (19.80,25.90)	22.70 ± 1.82 (20.30,26.00)	22.83 ± 1.65 (20.30,25.90)	22.37 ± 1.77 (19.40,26.00)

Data are the mean ± standard deviation (min-max), except sex (male/female) is the n/n. BMI, body mass index; SAD, single-ascending-dose; MD, multiple-dose.

### 3.2 Safety evaluations

Among the 60 subjects included in the SS, 16 subjects experienced a total of 31 TEAEs. None of the subjects received any concomitant medications during the entire study period. TEAEs by system organ class (SOC) and preferred term (PT) are summarized in Supplementary Table S2, whereas treatment-related adverse events (TRAEs) are summarized in Table 3. In the HZ-J001 group, 13 subjects reported 26 TEAEs, corresponding to an incidence of 26.0% (13/50), whereas 3 subjects in the placebo group experienced 5 TEAEs, with an incidence of 30.0% (3/10). TRAEs were reported in 8 subjects (9 events) in the HZ-J001 group (16.0%; 8/50) and in 2 subjects (4 events) of the placebo group (20.0%; 2/10). All TEAEs were grade 1 in severity except for one subject who experienced two grade 2 events in MD Cohort 5 (asthenia and dizziness). All TRAEs were grade 1 in severity. A total of 7 TEAEs in 4 subjects were lost to follow-up, while all other TEAEs resolved without medical intervention. No serious TEAEs were reported, and no subject discontinued the study due to TEAEs.

**TABLE 3:** Treatment- related adverse events sorted by SOC and PT.

SOC/PT	SAD cohort 1 (N = 10)		SAD cohort 2 (N = 10)		SAD cohort 3 (N = 10)		SAD cohort 4 (N = 10)		MD cohort 5 (N = 10)		Placebo (N = 10)		Total (N = 60)	
	Event	N (%)	Event	N (%)	Event	N (%)	Event	N (%)	Event	N (%)	Event	N (%)	Event	N (%)
Total	0	0 (0.00)	1	1 (10.00)	1	1 (10.00)	1	1 (10.00)	6	5 (50.00)	4	2 (20.00)	13	10 (16.67)
**Investigations**	0	0 (0.00)	1	1 (10.00)	1	1 (10.00)	1	1 (10.00)	1	1 (10.00)	0	0 (0.00)	4	4 (6.67)
Urine leukocytes positive	0	0 (0.00)	1	1 (10.00)	1	1 (10.00)	0	0 (0.00)	0	0 (0.00)	0	0 (0.00)	2	2 (3.33)
Blood bilirubin increased	0	0 (0.00)	0	0 (0.00)	0	0 (0.00)	0	0 (0.00)	1	1 (10.00)	0	0 (0.00)	1	1 (1.67)
Alanine aminotransferase increased	0	0 (0.00)	0	0 (0.00)	0	0 (0.00)	1	1 (10.00)	0	0 (0.00)	0	0 (0.00)	1	1 (1.67)
**General disorders and administration site conditions**	0	0 (0.00)	0	0 (0.00)	0	0 (0.00)	0	0 (0.00)	2	2 (20.00)	3	2 (20.00)	5	4 (6.67)
Application site pruritus	0	0 (0.00)	0	0 (0.00)	0	0 (0.00)	0	0 (0.00)	1	1 (10.00)	2	2 (20.00)	3	3 (5.00)
Application site rash	0	0 (0.00)	0	0 (0.00)	0	0 (0.00)	0	0 (0.00)	0	0 (0.00)	1	1 (10.00)	1	1 (1.67)
Application site papules	0	0 (0.00)	0	0 (0.00)	0	0 (0.00)	0	0 (0.00)	1	1 (10.00)	0	0 (0.00)	1	1 (1.67)
**Infections and infestations**	0	0 (0.00)	0	0 (0.00)	0	0 (0.00)	0	0 (0.00)	2	2 (20.00)	1	1 (10.00)	3	3 (5.00)
Application site folliculitis	0	0 (0.00)	0	0 (0.00)	0	0 (0.00)	0	0 (0.00)	2	2 (20.00)	1	1 (10.00)	3	3 (5.00)
**Skin and subcutaneous tissue disorders**	0	0 (0.00)	0	0 (0.00)	0	0 (0.00)	0	0 (0.00)	1	1 (10.00)	0	0 (0.00)	1	1 (1.67)
Skin dry	0	0 (0.00)	0	0 (0.00)	0	0 (0.00)	0	0 (0.00)	1	1 (10.00)	0	0 (0.00)	1	1 (1.67)

All treatment-related adverse events were grade 1 in severity. SOC, system organ class; PT, preferred term; SAD, single-ascending-dose; MD, multiple-dose.

In the local skin tolerability assessments, no abnormalities in either subjective symptoms or cutaneous findings were observed during the SAD phase. To evaluate cumulative local safety, maximum severity scores in each subject during the MD phase (MD Cohort 5) were analyzed (Supplementary Table S3). During the MD phase, 4 subjects (3 in the HZ-J001 group and 1 in the placebo group) reported mild local tolerability findings, comprising 3 cases of abnormal subjective symptoms and 3 cases of abnormal cutaneous findings. The highest subjective symptom score was 1 (mild, not interfere with daily activities or sleep), noted in 2 subjects (20.0%) in the HZ-J001 group and 1 subject in the placebo group. The highest objective cutaneous findings score was 2 (clearly visible erythema or slight edema, or mild papular response), observed in only 1 subject (10.0%) in the HZ-J001 group. None of these events led to study discontinuation, and all resolved without intervention, indicating favorable local tolerability of HZ-J001 cream.

The most frequently affected SOCs were investigations (20.0%; 12/60), general disorders and administration site conditions (6.7%; 4/60), and infections and infestations (5.0%; 3/60). In the HZ-J001 group, TEAEs reported in more than one subject included urine leukocytes positive (3 subjects, 3 events, 6.0%), heart rate decreased (3 subjects, 3 events, 6.0%), blood uric acid increased (3 subjects, 3 events, 6.0%), and application site folliculitis (2 subjects, 2 events, 4.0%). Heart rate decreased and blood uric acid increased were considered unlikely to be related to the study drug, and one case of urine leukocytes positive was also assessed as possibly unrelated. All other TEAEs in the HZ-J001 group were assessed as possibly or probably related to HZ-J001. In the placebo group, application site pruritus (2 subjects, 2 events, 20.0%) was the only TEAE reported in more than one subject, and was assessed as possibly related to placebo.

During the SAD phase, the incidence of TEAEs considered related to the study drug did not demonstrate a clear dose-dependent relationship. In addition, the overall incidence of TEAEs was comparable between the HZ-J001 and placebo groups. Overall, HZ-J001 cream at strengths of 1.0%, 1.5%, and 2.0%, applied to 20%–30% of BSA with a fixed amount of 3 mg cream/cm^2^, demonstrated a favorable safety and tolerability profile following both single and multiple administrations.

### 3.3 Pharmacokinetic characteristics

**FIGURE 2:**
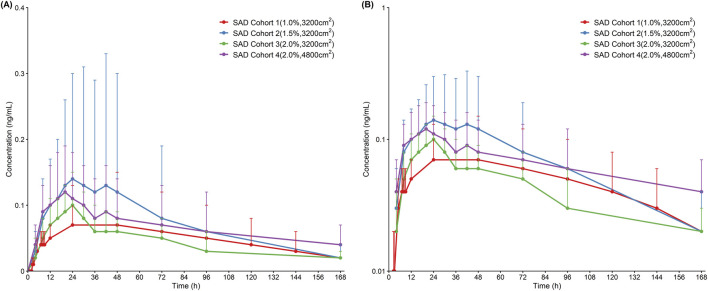
Mean plasma concentration-time profile of HZ-J001 after single-dose administration **(A)** Linear scale. **(B)** Semi-logarithmic scale. Data are presented as mean ± standard deviation (SD). SAD, single-ascending-dose.

Based on the mean plasma concentration-time profile (Figure 2), HZ-J001 cream demonstrated slow absorption following single topical application in healthy subjects. The overall concentration-time profile was relatively flat, indicating low systemic drug exposure. PK parameters of HZ-J001 after single-dose administration are summarized in Table 4. After single-dose administration, the C_max_ ranged from 0.09 to 0.16 ng/mL. The AUC ranged from 7.24 to 12.18 h·ng/mL for AUC_0-t_ and 10.76–16.80 h·ng/mL for AUC_0-∞_. The t_1/2_ was 54.00–103.57 h and T_max_ occurred approximately 16–24 h post-dose. All PK parameters demonstrated substantial inter-individual variability.

**TABLE 4:** Pharmacokinetic parameters of HZ-J001 after single-dose administration.

Parameters	SAD cohort 1	SAD cohort 2	SAD cohort 3	SAD cohort 4
C_max_ (ng/mL)	0.09 ± 0.06 (74.17) [N = 10]	0.16 ± 0.19 (116.81) [N = 10]	0.11 ± 0.05 (43.36) [N = 9]	0.14 ± 0.08 (54.82) [N = 10]
AUC_0-t_ (h·ng/mL)	7.79 ± 7.71 (98.96) [N = 10]	12.18 ± 13.96 (114.57) [N = 10]	7.24 ± 4.01 (55.41) [N = 9]	10.92 ± 8.40 (76.88) [N = 10]
AUC_0-∞_ (h·ng/mL)	11.73 ± 9.17 (78.20) [N = 8]	15.16 ± 15.03 (99.13) [N = 10]	10.76 ± 5.98 (55.59) [N = 9]	16.80 ± 12.80 (76.22) [N = 10]
AUC_Extrap_ (%)	22.34 ± 11.33 (50.73) [N = 8]	23.05 ± 12.33 (53.49) [N = 10]	28.29 ± 17.69 (62.54) [N = 9]	31.86 ± 12.85 (40.33) [N = 10]
T_max_(h)	17.99 (6.99–48.01,71.41) [N = 10]	20.00 (7.99–41.99,51.21) [N = 10]	24.00 (12.00–42.00,32.17) [N = 9]	16.00 (8.00–41.99,63.52) [N = 10]
λ_z_ (1/h)	0.01 ± 0.00 (33.87) [N = 8]	0.01 ± 0.00 (45.81) [N = 10]	0.01 ± 0.01 (70.05) [N = 9]	0.01 ± 0.00 (52.48) [N = 10]
t_1/2_(h)	54.00 ± 18.29 (33.88) [N = 8]	76.16 ± 31.46 (41.31) [N = 10]	92.09 ± 77.94 (84.64) [N = 9]	103.57 ± 50.15 (48.42) [N = 10]
MRT_0-t_(h)	52.97 ± 25.65 (48.42) [N = 10]	62.52 ± 9.83 (15.72) [N = 10]	59.12 ± 17.48 (29.57) [N = 9]	64.43 ± 14.25 (22.12) [N = 10]
MRT_0-∞_ (h)	98.41 ± 31.43 (31.94) [N = 8]	113.67 ± 39.50 (34.74) [N = 10]	137.63 ± 113.80 (82.68) [N = 9]	150.34 ± 72.10 (47.96) [N = 10]

Data are expressed as mean ± standard deviation (CV%), except for T_max_, which is presented as median (min-max, CV%). SAD, single-ascending-dose; C_max_, maximum concentration; AUC_0-t_, the area under the concentration versus time curve from zero to the last quantifiable concentration time; AUC_0-∞_, the area under the concentration versus time curve from zero to infinity; AUC_Extrap_, the area under the curve extrapolated percentage; T_max_, time to C_max_; λ_z_, elimination rate constant; t_1/2_, terminal elimination half-life; MRT_0-t_, mean residence time from zero to the last quantifiable concentration time; MRT_0-∞_, mean residence time from zero to infinity; CV, coefficient of variation.

Dose proportionality analyses were conducted for SAD Cohorts 1–4 (dose escalation), Cohorts 1–3 (strength escalation), and Cohorts 3–4 (application area escalation). As summarized in Supplementary Tables S4–S6, only the analysis for Cohorts 3–4 (application area escalation) showed that the estimated slopes (β) for C_max_, AUC_0-t_, and AUC_0-∞_ fell within the predefined decision intervals for linearity. In contrast, the corresponding slopes for Cohorts 1–4 (dose escalation) and Cohorts 1–3 (strength escalation) did not fall entirely within the predefined decision intervals. For all three analyses, the 90% CIs of the estimated slopes overlapped with the predefined decision intervals, precluding a definitive conclusion regarding dose proportionality. Moreover, the linear regression analyses yielded very low coefficients of determination (R^2^) across all parameters. This indicates substantial inter-subject variability and suggests weak evidence for dose proportionality. Consequently, these findings must be interpreted cautiously rather than implying a strong linear correlation between the administered dose and systemic exposure. Nevertheless, analysis across the SAD cohorts indicated that the fold increase in systemic exposure was less than the corresponding fold increase in dose. Evaluation of the effects of age, sex, and body weight on PK parameters revealed no statistically significant influence of age or sex. Body weight showed a statistically significant effect on T_max_ only in SAD Cohort 4 (Supplementary Table S7), with no significant effects observed on other parameters.

**FIGURE 3:**
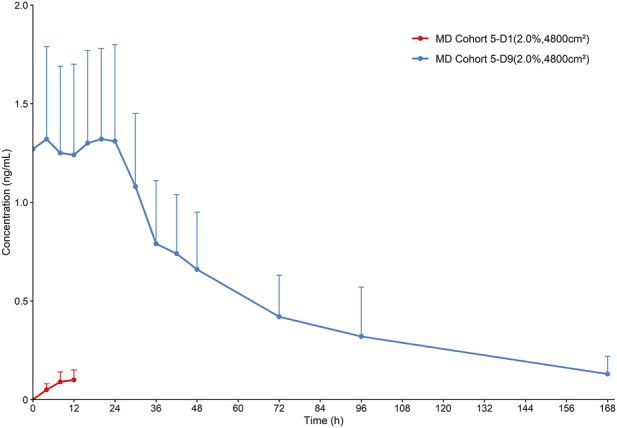
Mean plasma concentration-time profile of HZ-J001 after multiple-dose administration MD, multiple-dose.

The key PK parameters for the MD cohort (Cohort 5) are summarized in Table 5, and the corresponding mean plasma concentration-time profiles are presented in Figure 3. It should be noted that, owing to the dosing interval, PK parameters following the initial administration presented in Table 5 (C_max_, AUC_0-τ_, and T_max_) were calculated based solely on plasma concentration data collected within the first 12 h post-dose and therefore do not fully characterize the complete systemic exposure profile of a single topical dose. Compared with single-dose administration, MD resulted in an earlier T_max_ at steady state (T_ss,max_ = 4 h) and was associated with drug accumulation. The C_ss,av_ was 1.27 ng/mL and AUC_ss,0-τ_ was 15.30 h·ng/mL. The R_ac_ values for C_max_ and AUC_0-τ_ were 15.71 and 28.80, respectively. Although the rate and extent of absorption increased after MD, the overall systemic exposure to HZ-J001 remained low. Regression analysis of trough concentrations collected prior to the morning doses on Days 7, 8, and 9 showed no statistically significant differences (P > 0.05), indicating that steady state was achieved by Day 7 (Supplementary Figure S1).

**TABLE 5:** Pharmacokinetic parameters of HZ-J001 in multiple-dose Cohort five.

Pharmacokinetic parameters	Mean ± standard deviation (CV%) (N = 10)
Initial administration	
C_max_ (ng/mL)	0.10 ± 0.05 (50.82)
AUC_0-τ_ (h·ng/mL)	0.75 ± 0.41 (55.20)
T_max_ (h)	11.97 (8.00–11.97,17.78)
Final administration	
C_ss,min_ (ng/mL)	1.20 ± 0.45 (37.29)
C_ss,max_ (ng/mL)	1.35 ± 0.45 (33.22)
C_ss,av_ (ng/mL)	1.27 ± 0.45 (35.08)
T_ss,max_ (h)	4.00 (0.00–4.00,86.07)
AUC_ss,0-τ_ (h·ng/mL)	15.30 ± 5.36 (35.06)
DF (%)	13.49 ± 6.71 (49.71)
R_ac_ of C_max_	15.71 ± 10.02 (63.78)
R_ac_ of AUC_0-τ_	28.80 ± 25.46 (88.43)

Data are expressed as mean ± standard deviation (CV%), except for T_max_ and T_ss,max_, which are presented as median (min-max, CV%). CV, coefficient of variation; C_max_, maximum concentration; AUC_0-τ_, the area under the concentration versus time curve during the dosing interval; T_max_, time to C_max_; C_ss,min_, minimum steady-state concentrations; C_ss,max_, maximum steady-state concentrations; C_ss,av_, average steady-state concentration; T_ss,max_, time to C_ss,max_; AUC_ss, 0-τ_, the area under the concentration versus time curve during the dosing interval at steady state; DF, degree of fluctuation; R_ac_, accumulation ratio.

## 4 Discussion

This study represents the first clinical evaluation of the safety, tolerability, and PK characteristics of HZ-J001 cream in healthy subjects. The results demonstrated that HZ-J001 cream, at strengths ranging from 1.0% to 2.0% and applied at a fixed amount of 3 mg cream/cm^2^ over 20%–30% BSA, was generally safe and well tolerated, with consistently low systemic exposure.

AD often presents in early childhood, typically within the first 3–6 months of life, and its prevalence is higher in children than in adults (Mazgaj et al., 2025). Given the poor adherence to oral medications among pediatric patients, topical therapies are particularly advantageous in this population. Moreover, systemic administration is associated with significantly higher risks of systemic AEs compared to topical delivery (Simpson et al., 2016; Ruzicka et al., 2017). Therefore, topical formulations with low systemic exposure may offer an improved safety profile (Bissonnette et al., 2016). Local JAK inhibitors have emerged as promising options for AD therapy. However, clinical data on topical tofacitinib remain limited, highlighting the need for continued development of novel topical JAK-targeted agents. The development of HZ-J001 cream was driven by modification of the crystalline form of tofacitinib citrate into a tartrate salt, coupled with strategic optimizations in indication, formulation, route of administration, and manufacturing process, collectively conferring favorable local PK properties. This formulation strategy was designed to enhance drug retention at the affected skin sites, improving local efficacy while minimizing systemic exposure and the associated risks of systemic AEs.

Although clinical experience with topical tofacitinib is more limited than that of approved topical JAK inhibitors (e.g., ruxolitinib and delgocitinib), several studies have demonstrated the safety and feasibility of achieving clinical activity. A 4-week phase IIa randomized trial evaluated 2% tofacitinib ointment applied twice daily in adults with mild-to-moderate AD and reported significant efficacy with an acceptable short-term safety profile (Bissonnette et al., 2016). In plaque psoriasis, a multicenter phase IIa study also assessed topical tofacitinib ointment formulations over 4 weeks and showed clinical improvement with favorable local tolerability (Ports et al., 2013). Importantly, these published studies were conducted exclusively in patient populations and typically involved limited treated BSA corresponding to disease lesions, rather than standardized large-area application. Building upon these earlier findings, the present study was specifically designed to systematically evaluate the safety, tolerability, and systemic PK of a novel optimized formulation, HZ-J001 cream, in healthy volunteers under relatively large treated-area conditions (20%–30% BSA). This design provides a conservative and clinically informative assessment of systemic exposure and short-term tolerability, thereby complementing existing patient-based evidence and supporting the further clinical development of topical tofacitinib formulations.

In the present study, C_max_ values ranged from 0.09 to 0.16 ng/mL following single-dose administration, whereas a steady-state C_ss,max_ of only 1.35 ng/mL was observed after MD. These findings are consistent with previously reported data for topical tofacitinib. In a phase IIa study of patients with AD treated with topical tofacitinib ointment for 4 weeks, the maximum observed plasma concentration was 2.72 ng/mL (Purohit et al., 2019). In contrast, C_max_ values as high as 71.28 ng/mL have been reported in healthy Chinese subjects receiving a single 5 mg oral dose of tofacitinib (Xeljanz®) (Xu et al., 2022). Taken together, these comparisons indicate that systemic exposure following topical HZ-J001 administration (<2 ng/mL) is substantially lower than that observed with oral dosing, supporting the potential of topical delivery to achieve local therapeutic effects while minimizing systemic exposure.

Despite the low systemic exposure observed in the present study, topical administration is expected to achieve substantially higher drug concentrations within the skin. Previous studies have demonstrated that topical JAK inhibitors can produce high local concentrations in the epidermis and upper dermis while maintaining minimal systemic absorption (Papp et al., 2021; Nakashima et al., 2022). This PK profile is thought to underlie their clinical efficacy in inflammatory skin diseases such as AD, psoriasis, and vitiligo, despite low plasma concentrations (Miot et al., 2023), and supports effective local target engagement with a reduced risk of systemic AEs. In addition, preclinical studies of HZ-J001 cream demonstrated favorable local PK characteristics following topical administration, characterized by enhanced drug accumulation at the site of application, which is critical for achieving sufficient local therapeutic exposure. Furthermore, evidence suggests that oral administration may not achieve sufficiently high drug concentrations in the skin to effectively inhibit local JAK-STAT signaling pathways, potentially resulting in inferior therapeutic effects compared with direct topical application (Böll et al., 2025). Although drug concentrations in the skin were not directly measured in the present study, the totality of existing clinical and preclinical evidence supports the expectation that topical HZ-J001 achieves adequate local exposure to enable effective target engagement.

Following topical administration of HZ-J001 cream, the plasma concentration-time profiles were relatively flat and systemic absorption was relatively slow, as reflected by a T_max_ of 16–24 h after single dosing. In addition, the prolonged t_1/2_ and mean residence time (MRT) further suggest drug retention within the skin. This PK profile is likely attributable to the tartrate salt modification, which was specifically designed to enhance local drug residence time in lesional skin and may support more sustained therapeutic activity. Dose proportionality analysis further revealed that the relative increase in systemic exposure was consistently less than the corresponding increase in dose, suggesting a trend toward saturation of percutaneous absorption. Similar findings have been documented for other topical formulations (Gong et al., 2021). Within the studied dose range and application area, transdermal absorption appears to approach an upper limit. While this may complicate the establishment of a linear dose-exposure relationship, it also indicates that systemic exposure does not increase proportionally with higher topical doses, thereby conferring a potential safety advantage during dose escalation.

Substantial inter-individual variability was observed across all key PK parameters in this study. This finding is consistent with reports from early-phase clinical trials of other topical formulations, where such variability is common (Gong et al., 2021). The observed variability likely stems from the inherent complexity of transdermal delivery, a process influenced by a multitude of physiological and physical factors. These include individual differences in skin barrier integrity, site-specific skin properties, variations in application thickness, and differences in rubbing intensity during administration (Stoughton, 1989; Wiechers, 1989; Singh and Morris, 2011; Konda et al., 2012). Furthermore, the extremely low systemic exposure levels measured in this study may have amplified the relative variability in PK parameters. For drugs with low bioavailability, high relative variability in exposure parameters is an anticipated PK characteristic (Daublain et al., 2017).

Following the collection of trough plasma samples before the morning dose on Days 7, 8, and 9 from the MD Cohort 5, regression analysis indicated that the drug had reached steady state after 7 days of continuous administration. The R_ac_ values calculated after 9 days of dosing revealed a certain degree of accumulation, with R_ac_ values of 15.71 for C_max_ and 28.80 for AUC_0-τ_. Compared with single-dose administration, the T_ss,max_ (4 h) occurred earlier and systemic exposure increased, indicating an enhancement in both the rate and extent of drug absorption after repeated administration. This accumulation can be primarily attributed to the intrinsic PK properties of the drug. Following a single dose, HZ-J001 cream is absorbed slowly, with a mean T_max_ of 16–24 h and a mean t_1/2_ ranging from 54.00 to 103.57 h. Consequently, the 12-h dosing interval was insufficient for complete elimination of the previous dose in MD Cohort 5, thereby leading to gradual accumulation. Nevertheless, given the extremely low baseline exposure after the first dose, overall systemic exposure remained low even after accumulation and was substantially lower than that observed with oral tofacitinib (Xu et al., 2022).

A statistically significant effect of body weight on T_max_ was uniquely observed in SAD Cohort 4. However, the clinical relevance of this isolated finding is questionable, and it must be interpreted with caution. First, this analysis was based on a very small sample size (n = 5 per group), which makes the results unreliable. The high CV% for T_max_ in this cohort (63.52%) indicates substantial intrinsic variability in this parameter. This pronounced variance is driven by inherent individual physiological differences. The rate of percutaneous absorption is profoundly influenced by local skin characteristics, such as regional skin thickness (particularly the stratum corneum) and skin hydration levels (Dąbrowska et al., 2018). Furthermore, cutaneous blood supply and microcirculation play a pivotal role and the variations in local skin perfusion can significantly impact the clearance rate of the drug from the dermis into the systemic circulation, thereby directly altering the observed T_max_ (Roberts et al., 2021). Moreover, given that the application BSA was identical for all subjects in SAD Cohort 4, the biological plausibility of a body weight-dependent effect on the rate of absorption is low. Importantly, body weight showed no significant influence on T_max_ across other cohorts. Therefore, this isolated statistical significance for T_max_ is likely incidental and does not represent a clinically meaningful relationship.

It is important to note a specific methodological limitation regarding the characterization of the terminal elimination phase. In our NCA results of the SAD cohorts, the area under the curve extrapolated percentage (AUC_Extrap_) frequently exceeded the conventional 20% threshold, ranging from 22.34% to 31.86%. This high extrapolation percentage is a recognized and common challenge in the PK evaluation of topically applied dermatological products with extremely low systemic bioavailability. Specifically, due to the slow and sustained transdermal absorption coupled with C_max_ already hovering near the LLOQ (0.01 ng/mL), plasma concentrations during the terminal phase frequently fell below the quantifiable threshold prematurely. Correspondingly, this limits the precision and interpretability of the extrapolated parameters, including t_1/2_, AUC_0-∞_, mean residence time from zero to infinity (MRT_0-∞_) and elimination rate constant (λ_z_), which should be interpreted cautiously and are preferably considered as exploratory estimates rather than definitive values.

In this study, a fixed treated BSA (3,200–4,800 cm^2^) with standardized application amount of 3 mg cream/cm^2^ was used instead of dosing normalized to individual BSA. This strategy reflects the characteristics of topical dermatologic products, for which the effective dose is primarily determined by the amount applied per unit area (mg/cm^2^) and the area of the treated skin surface, rather than by systemic parameters such as body size (Patel et al., 2024). Percutaneous absorption of topical agents is governed mainly by local skin and formulation-related factors, and normalization by individual BSA does not meaningfully reduce variability in systemic exposure. Accordingly, fixed-area dosing with a standardized application amount is commonly adopted in early-phase clinical studies of topical products to ensure consistent dose delivery and to characterize systemic exposure under conditions expected to result in relatively high topical drug exposure (Li et al., 2023). Therefore, a fixed amount of 3 mg cream/cm^2^ and a predefined treated BSA range of 3,200–4,800 cm^2^ were selected in the present study.

Oral JAK inhibitors are associated with AEs such as nasopharyngitis, headache, upper respiratory tract infections, and nausea (Zhang et al., 2021), and carry U.S. FDA black box warnings for increased risks of malignancy, serious infections, major adverse cardiovascular events, and thrombosis (Mazgaj et al., 2025). In clinical studies of 2% topical tofacitinib ointment for mild-to-moderate AD, the most commonly reported TEAEs were infections and infestations, as well as application-site reactions such as pain and pruritus (Bissonnette et al., 2016). In the present study, the most frequently observed SOCs were investigations, including heart rate decreased, blood uric acid increased, blood bilirubin increased, and urine leukocytes positive. Heart rate decreased was more frequently observed in subjects with lower baseline heart rates and was likely related to reduced physical activity during confinement. Blood uric acid increased and blood bilirubin increased were difficult to attribute to pharmacological effects given the extremely low systemic exposure. Nevertheless, given that urinary tract infections have been reported with oral tofacitinib treatment (Xu et al., 2022), the potential association between HZ-J001 and urine leukocytes positive cannot be entirely excluded and will continue to be closely monitored in subsequent studies.

Other AEs of interest included application-site reactions (e.g., application site pruritus) and infections (e.g., application site folliculitis), consistent with the safety profiles reported for other topical JAK inhibitors, including ruxolitinib cream (Zhou et al., 2024) and delgocitinib ointment (Nakagawa et al., 2020). The occurrence of folliculitis and pruritus at application sites may have been partially influenced by changes in the local microenvironment due to prolonged occlusion, limited bathing during the study period, and hot weather conditions. This finding suggests that appropriate local hygiene and skin care may further reduce the risk of such reactions in clinical practice. Systemic exposure may be higher in patients with AD, who typically have impaired skin barrier function, compared with healthy subjects with intact skin barriers. Therefore, continued monitoring of these safety signals in subsequent patient studies is warranted. Overall, the safety findings indicate that HZ-J001 cream has an acceptable safety profile in healthy subjects, with TEAEs consistent with the expected risk spectrum of topical JAK inhibitors. These results further support the fundamental advantage of topical delivery in minimizing systemic exposure and avoiding the systemic risks associated with oral JAK inhibitors, thereby providing a strong safety foundation for further clinical development in patients with AD.

This study has several limitations. First, although the sample size was considered appropriate for a phase Ia clinical trial, it may have limited the ability to detect rare or low-incidence AEs and was associated with substantial inter-individual variability. Second, due to the practical challenges of recruiting female volunteers for early-phase clinical trials, the study population was predominantly male (52 males vs. 8 females). While our preliminary analyses did not indicate a statistically significant effect of sex on PK parameters, the limited number of female participants restricts the generalizability of these findings across genders. Third, due to the inherent design of this early-phase dose-escalation trial, only two subjects per cohort were randomized to receive the placebo. This small intra-cohort placebo sample size precluded robust direct statistical comparisons between the active treatment and placebo groups within each individual cohort. Consequently, safety evaluations were primarily descriptive and relied on pooled placebo data. Finally, all participants were healthy subjects with an intact skin barrier, which may have resulted in lower systemic exposure compared with patients with AD, and no efficacy data were collected. Consequently, larger-scale clinical trials in the target patient population are required to comprehensively characterize the safety profile and systemic exposure of HZ-J001 cream under disease conditions.

## 5 Conclusion

Topical administration of HZ-J001 cream at strengths of 1.0%–2.0%, with a standardized application amount of 3 mg cream/cm^2^ and an application area of 20%–30% BSA, was safe and well tolerated in healthy subjects. Following single-dose application, plasma concentration-time profiles were generally flat, indicating low systemic exposure and a low potential risk of systemic TEAEs. Dose proportionality for C_max_, AUC_0-t_, and AUC_0-∞_ across single-dose groups was inconclusive, with systemic exposure increasing to a lesser extent than the corresponding dose escalation. Compared with single dosing, MD increased the rate and extent of absorption and overall systemic exposure remained low. Given the differences in skin barrier function between healthy subjects and patients, further clinical studies are warranted to evaluate the safety, PKs, and efficacy of HZ-J001 cream in patients with AD and other intended dermatologic indications.

## Data Availability

The datasets presented in this article are not readily available because the data, trial protocol and statistical analysis supporting this study were obtained from the corresponding author, which are not publicly available due to privacy and ethical constraints. Requests to access the datasets should be directed to Chengxian Guo (gchxyy@163.com).
