# Supplementary material for: A randomized controlled phase Ia clinical trial to evaluate the safety, tolerability and pharmacokinetic characteristics of tofacitinib tartrate cream (HZ-J001) in healthy Chinese subjects
Source: Front Pharmacol. 2026 May 7;17:1821901. doi: 10.3389/fphar.2026.1821901 (PMC13190169; doi:10.3389/fphar.2026.1821901)

**Supplementary materials**

Table S1 The detailed grading criteria for local skin tolerability assessment

| Characteristics | Score |
| --- | --- |
| Subjective symptoms (pruritus, pain, or burning sensation) | |
| None | 0 |
| Mild, not interfere with daily activities or sleep | 1 |
| Moderate, interferes with daily activities but not sleep | 2 |
| Severe, interferes with sleep | 3 |
| Objective cutaneous findings | |
| No evidence of irritation | 0 |
| Minimal erythema, barely perceptible | 1 |
| Clearly visible erythema or slight edema, or mild papular response | 2 |
| Erythema and papules | 3 |
| Clearly evident edema | 4 |
| Erythema, edema, and papules | 5 |
| Vesicular eruption | 6 |
| Strong reaction spreading beyond the application site | 7 |

Table S2 Treatment-emergent adverse events sorted by SOC and PT

| SOC/PT | SAD Cohort 1 (N=10) | | SAD Cohort 2 (N=10) | | SAD Cohort 3 (N=10) | | SAD Cohort 4 (N=10) | | MD Cohort 5 (N=10) | | Placebo (N=10) | | Total (N=60) | |
| --- | --- | --- | --- | --- | --- | --- | --- | --- | --- | --- | --- | --- | --- | --- |
|  | Event | N (%) | Event | N (%) | Event | N (%) | Event | N (%) | Event | N (%) | Event | N (%) | Event | N (%) |
| Total | 1 | 1 (10.00) | 4 | 1(10.00) | 2 | 1(10.00) | 4 | 4 (40.00) | 15 | 6 (60.00) | 5 | 3 (30.00) | 31 | 16 (26.67) |
| **Investigations** | 0 | 0(0.00) | 3 | 1(10.00) | 2 | 1 (10.00) | 4 | 4 (40.00) | 8 | 5 (50.00) | 1 | 1 (10.00) | 18 | 12 (20.00) |
| Urine leukocytes positive | 0 | 0(0.00) | 1 | 1(10.00) | 1 | 1 (10.00) | 0 | 0(0.00) | 1 | 1 (10.00) | 0 | 0(0.00) | 3 | 3(5.00) |
| Heart rate decreased | 0 | 0(0.00) | 0 | 0(0.00) | 0 | 0(0.00) | 0 | 0(0.00) | 3 | 3 (30.00) | 0 | 0(0.00) | 3 | 3(5.00) |
| Blood uric acid increased | 0 | 0(0.00) | 0 | 0(0.00) | 0 | 0(0.00) | 1 | 1(10.00) | 2 | 2 (20.00) | 0 | 0(0.00) | 3 | 3(5.00) |
| Blood bilirubin increased | 0 | 0(0.00) | 0 | 0(0.00) | 0 | 0(0.00) | 0 | 0(0.00) | 1 | 1 (10.00) | 1 | 1(10.00) | 2 | 2(3.33) |
| Alanine aminotransferase increased | 0 | 0(0.00) | 0 | 0(0.00) | 0 | 0(0.00) | 1 | 1(10.00) | 0 | 0(0.00) | 0 | 0(0.00) | 1 | 1(1.67) |
| Protein urine present | 0 | 0(0.00) | 0 | 0(0.00) | 1 | 1(10.00) | 0 | 0(0.00) | 0 | 0(0.00) | 0 | 0(0.00) | 1 | 1 (1.67) |
| Squamous epithelial cells urine increased | 0 | 0(0.00) | 1 | 1 (10.00) | 0 | 0(0.00) | 0 | 0(0.00) | 0 | 0(0.00) | 0 | 0(0.00) | 1 | 1 (1.67) |
| Occult blood urine positive | 0 | 0(0.00) | 0 | 0(0.00) | 0 | 0(0.00) | 0 | 0(0.00) | 1 | 1 (10.00) | 0 | 0(0.00) | 1 | 1 (1.67) |
| Bacteria test positive | 0 | 0(0.00) | 1 | 1 (10.00) | 0 | 0(0.00) | 0 | 0(0.00) | 0 | 0(0.00) | 0 | 0(0.00) | 1 | 1 (1.67) |
| Electrocardiogram T wave abnormal | 0 | 0(0.00) | 0 | 0(0.00) | 0 | 0(0.00) | 1 | 1(10.00) | 0 | 0(0.00) | 0 | 0(0.00) | 1 | 1 (1.67) |
| Blood glucose decreased | 0 | 0(0.00) | 0 | 0(0.00) | 0 | 0(0.00) | 1 | 1(10.00) | 0 | 0(0.00) | 0 | 0(0.00) | 1 | 1 (1.67) |
| **General disorders and administration site conditions** | 0 | 0(0.00) | 0 | 0(0.00) | 0 | 0(0.00) | 0 | 0(0.00) | 3 | 2(20.00) | 3 | 2 (20.00) | 6 | 4 (6.67) |
| Application site pruritus | 0 | 0(0.00) | 0 | 0(0.00) | 0 | 0(0.00) | 0 | 0(0.00) | 1 | 1(10.00) | 2 | 2 (20.00) | 3 | 3 (5.00) |
| Application site rash | 0 | 0(0.00) | 0 | 0(0.00) | 0 | 0(0.00) | 0 | 0(0.00) | 0 | 0(0.00) | 1 | 1 (10.00) | 1 | 1 (1.67) |
| Application site papules | 0 | 0(0.00) | 0 | 0(0.00) | 0 | 0(0.00) | 0 | 0(0.00) | 1 | 1(10.00) | 0 | 0(0.00) | 1 | 1 (1.67) |
| Asthenia | 0 | 0(0.00) | 0 | 0(0.00) | 0 | 0(0.00) | 0 | 0(0.00) | 1 | 1(10.00) | 0 | 0(0.00) | 1 | 1 (1.67) |
| **Infections and infestations** | 0 | 0(0.00) | 0 | 0(0.00) | 0 | 0(0.00) | 0 | 0(0.00) | 2 | 2(20.00) | 1 | 1 (10.00) | 3 | 3 (5.00) |
| Application site folliculitis | 0 | 0(0.00) | 0 | 0(0.00) | 0 | 0(0.00) | 0 | 0(0.00) | 2 | 2(20.00) | 1 | 1 (10.00) | 3 | 3 (5.00) |
| **Nervous system disorders** | 0 | 0(0.00) | 0 | 0(0.00) | 0 | 0(0.00) | 0 | 0(0.00) | 1 | 1(10.00) | 0 | 0(0.00) | 1 | 1 (1.67) |
| Dizziness | 0 | 0(0.00) | 0 | 0(0.00) | 0 | 0(0.00) | 0 | 0(0.00) | 1 | 1(10.00) | 0 | 0(0.00) | 1 | 1 (1.67) |
| **Skin and subcutaneous tissue disorders** | 0 | 0(0.00) | 0 | 0(0.00) | 0 | 0(0.00) | 0 | 0(0.00) | 1 | 1(10.00) | 0 | 0(0.00) | 1 | 1 (1.67) |
| Skin dry | 0 | 0(0.00) | 0 | 0(0.00) | 0 | 0(0.00) | 0 | 0(0.00) | 1 | 1(10.00) | 0 | 0(0.00) | 1 | 1 (1.67) |
| **Renal and urinary disorders** | 0 | 0(0.00) | 1 | 1 (10.00) | 0 | 0(0.00) | 0 | 0(0.00) | 0 | 0(0.00) | 0 | 0(0.00) | 1 | 1 (1.67) |
| Urine abnormality | 0 | 0(0.00) | 1 | 1 (10.00) | 0 | 0(0.00) | 0 | 0(0.00) | 0 | 0(0.00) | 0 | 0(0.00) | 1 | 1 (1.67) |
| **Cardiac disorders** | 1 | 1 (10.00) | 0 | 0(0.00) | 0 | 0(0.00) | 0 | 0(0.00) | 0 | 0(0.00) | 0 | 0(0.00) | 1 | 1 (1.67) |
| Atrial escape rhythm | 1 | 1 (10.00) | 0 | 0(0.00) | 0 | 0(0.00) | 0 | 0(0.00) | 0 | 0(0.00) | 0 | 0(0.00) | 1 | 1 (1.67) |

For continuous variables, abnormal fluctuations were recorded as treatment-emergent adverse events when the values fell strictly outside the normal reference range and were evaluated as clinically significant by the investigator. All treatment-emergent adverse events were grade 1 in severity except for one subject who experienced two grade 2 events in MD Cohort 5 (asthenia and dizziness). SOC, system organ class; PT, preferred term; SAD, single-ascending-dose; MD, multiple-dose.

Table S3 Summary of maximum severity scores in each subject for local skin tolerability in the multiple-dose Cohort 5

| Score | HZ-J001 group (N=10) | | Placebo group (N=2) | | Total (N=12) | |
| --- | --- | --- | --- | --- | --- | --- |
|  | Subjective symptoms | Objective cutaneous findings | Subjective symptoms | Objective cutaneous findings | Subjective symptoms | Objective cutaneous findings |
| 0 | 8(80.00) | 8(80.00) | 1(50.00) | 1(50.00) | 9(75.00) | 9(75.00) |
| 1 | 2(20.00) | 1(10.00) | 1(50.00) | 1(50.00) | 3(25.00) | 2(16.67) |
| 2 | 0(0.00) | 1(10.00) | 0(0.00) | 0(0.00) | 0(0.00) | 1(8.33) |
| 3 | 0(0.00) | 0(0.00) | 0(0.00) | 0(0.00) | 0(0.00) | 0(0.00) |
| 4 | / | 0(0.00) | / | 0(0.00) | / | 0(0.00) |
| 5 | / | 0(0.00) | / | 0(0.00) | / | 0(0.00) |
| 6 | / | 0(0.00) | / | 0(0.00) | / | 0(0.00) |
| 7 | / | 0(0.00) | / | 0(0.00) | / | 0(0.00) |

Data are the N (percentage).

Table S4 Linear analysis of pharmacokinetic parameters after single dose administration (dose escalation: SAD Cohort 1-4)

| Parameter | Regression coefficient (β) and 90% CI | R² | Decision interval |
| --- | --- | --- | --- |
| C_max_ | 0.49(0.06,0.92) | 0.0915 | (0.68,1.33) |
| AUC_0-t_ | 0.59(−0.03,1.21) | 0.0656 | (0.80,1.20) |
| AUC_0-∞_ | 0.34(−0.21,0.90) | 0.0306 | (0.80,1.20) |

The decision interval was constructed using a log-transformed power model: ln(PK)=β_0_+β×ln(Dose)+ε. The standard interval for linearity is β_L_-β_U_, Where β_L_=1+lnθ_L_/ln_γ_, β_U_=1+lnθ_U_/ln_γ_,and γ= highest dose/ lowest dose. For AUC_0-t_ and AUC_0-∞_, θ_L=_0.8 and θ_U_=1.25. For C_max_, θ_L_=0.7 and θ_U_=1.43. SAD, single-ascending-dose; C_max_, maximum concentration; AUC_0-t_, the area under the concentration versus time curve from zero to the last quantifiable concentration time; AUC_0-∞_, the area under the concentration versus time curve from zero to infinity; CI, confidence interval; R², coefficient of determination.

Table S5 Linear analysis of pharmacokinetic parameters after single dose administration (strength escalation: SAD Cohort 1-3)

| Parameter | Regression coefficient (β) and 90% CI | R² | Decision interval |
| --- | --- | --- | --- |
| C_max_ | 0.13(−0.52,0.78) | 0.0043 | (0.49,1.52) |
| AUC_0-t_ | 0.13(−0.68,0.95) | 0.0029 | (0.68,1.32) |
| AUC_0-∞_ | 0.31(−0.52,1.15) | 0.0149 | (0.68,1.32) |

The decision interval was constructed using a log-transformed power model: ln(PK)=β_0_+β×ln(Dose)+ε. The standard interval for linearity is β_L_-β_U_, Where β_L_=1+lnθ_L_/ln_γ_, β_U_=1+lnθ_U_/ln_γ_,and γ= highest dose/ lowest dose. For AUC_0-t_ and AUC_0-∞_, θ_L=_0.8 and θ_U_=1.25. For C_max_, θ_L_=0.7 and θ_U_=1.43. SAD, single-ascending-dose; C_max_, maximum concentration; AUC_0-t_, the area under the concentration versus time curve from zero to the last quantifiable concentration time; AUC_0-∞_, the area under the concentration versus time curve from zero to infinity; CI, confidence interval; R², coefficient of determination.

Table S6 Linear analysis of pharmacokinetic parameters after single dose administration (application area escalation: SAD Cohort 3-4)

| Parameter | Regression coefficient (β) and 90% CI | R² | Decision interval |
| --- | --- | --- | --- |
| C_max_ | 0.56(−0.37,1.49) | 0.0611 | (0.12,1.88) |
| AUC_0-t_ | 0.91(−0.41,2.23) | 0.0780 | (0.45,1.55) |
| AUC_0-∞_ | 0.99(−0.47,2.45) | 0.0753 | (0.45,1.55) |

The decision interval was constructed using a log-transformed power model: ln(PK)=β_0_+β×ln(Dose)+ε. The standard interval for linearity is β_L_-β_U_, Where β_L_=1+lnθ_L_/ln_γ_, β_U_=1+lnθ_U_/ln_γ_,and γ= highest dose/ lowest dose. For AUC_0-t_ and AUC_0-∞_, θ_L=_0.8 and θ_U_=1.25. For C_max_, θ_L_=0.7 and θ_U_=1.43. SAD, single-ascending-dose; C_max_, maximum concentration; AUC_0-t_, the area under the concentration versus time curve from zero to the last quantifiable concentration time; AUC_0-∞_, the area under the concentration versus time curve from zero to infinity; CI, confidence interval; R², coefficient of determination.

Table S7 Nonparametric analysis of the influence of body weight on pharmacokinetic parameters in SAD Cohort 4 (N=10)

| Parameter | Statistical description | ＞63.45kg | ≤63.45kg |
| --- | --- | --- | --- |
| T_max_(h) | n | 5 | 5 |
|  | Mean (SD) | 10.40(5.36) | 23.60(11.17) |
|  | Median (Q1, Q3) | 8.00(8.00,8.00) | 20.00(19.99,24.01) |
|  | Min, Max | 8.00,19.99 | 12.00,41.99 |
|  | Rank Sum Test | 17.00 | |
|  | P value | 0.0361 | |
| C_max_(ng/mL) | n | 5 | 5 |
|  | Mean (SD) | 0.09(0.03) | 0.19(0.09) |
|  | Median (Q1, Q3) | 0.08(0.07,0.12) | 0.21(0.12,0.25) |
|  | Min, Max | 0.07,0.13 | 0.08,0.28 |
|  | Rank Sum Test | 19.00 | |
|  | P value | 0.0947 | |
| AUC0-t(h·ng/mL) | n | 5 | 5 |
|  | Mean (SD) | 6.66(3.08) | 15.19(10.19) |
|  | Median (Q1, Q3) | 7.26(5.04,7.71) | 12.17(10.43,14.27) |
|  | Min, Max | 2.54,10.76 | 6.39,32.66 |
|  | Rank Sum Test | 19.00 | |
|  | P value | 0.0947 | |
| AUC_0-∞_(h·ng/mL) | n | 5 | 5 |
|  | Mean (SD) | 11.82(8.69) | 21.78(15.21) |
|  | Median (Q1, Q3) | 9.73(7.69,12.47) | 16.95(12.20,22.41) |
|  | Min, Max | 3.10,26.12 | 9.75,47.57 |
|  | Rank Sum Test | 21.00 | |
|  | P value | 0.2101 | |

The median body weight in SAD Cohort 4 was 63.45 kg. SAD, single-ascending-dose; C_max_, maximum concentration; T_max_, time to C_max_; AUC_0-t_, the area under the concentration versus time curve from zero to the last quantifiable concentration time; AUC_0-∞_, the area under the concentration versus time curve from zero to infinity; SD, standard deviation; Q1, first quartile; Q3, third quartile.

Figure S1 Mean trough plasma concentration-time profile of HZ-J001 during the Day 7–9 in multiple-dose Cohort 5


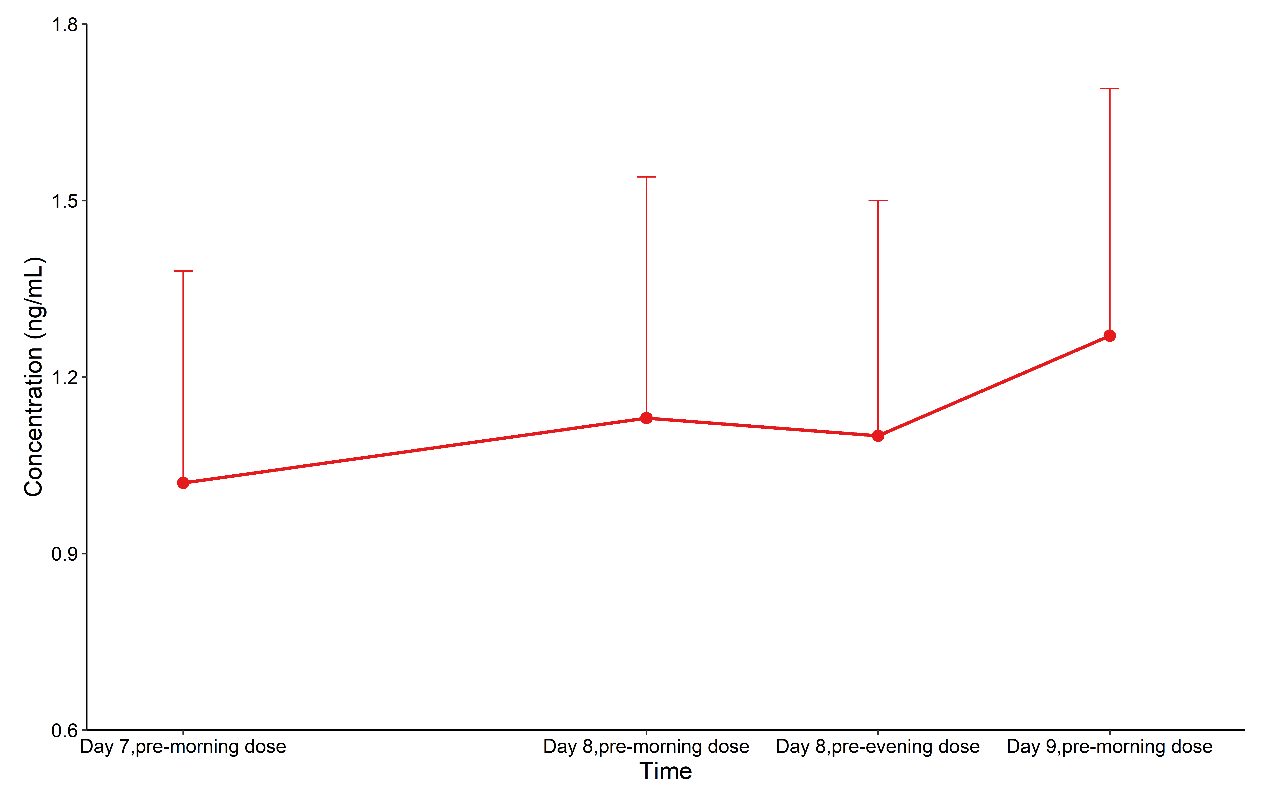

Supplement: Supplementary file 1 [file Supplementaryfile1.docx]
